# PROGRAM CHARACTERISTICS ASSOCIATED WITH REDUCED FEAR OF FALLING: A SYSTEMATIC REVIEW AND META-ANALYSIS OF RCTS

**DOI:** 10.1093/geroni/igz038.070

**Published:** 2019-11-08

**Authors:** Marlot Kruisbrink, Kim Delbaere, Gertrudis Kempen, Rik Crutzen, G A Rixt Zijlstra

**Affiliations:** 1 Maastricht University, Care and Public Health Research Institute (CAPHRI), Department of Health Services Research, Maastricht, Netherlands; 2 Neuroscience Research Australia, School of Public Health and Community Medicine, UNSW, Randwick, New South Wales, Australia

## Abstract

Fear of falling (FOF) is common among older people and can result in activity avoidance and decreased physical functioning. Different types of interventions have demonstrated significant small reductions in FOF. To optimize effect sizes, we sought to identify characteristics of interventions that were associated with a change in FOF. Five scientific databases were searched for articles using randomized controlled trial designs in community-dwelling older people without medical conditions. Data extraction included intervention type, setting, group format, type of supervision, provider, delivery format, duration, number of sessions, contact time, and risk of bias (assessed with the Cochrane Collaboration’s Risk of Bias Tool). After screening of titles, abstracts, and full texts, 55 unique studies – reporting on 68 interventions – were systematically reviewed. The majority of interventions focused on exercise (n=50). Interventions were performed at home (n=21) or in a community setting (n=23), were delivered in a group (n=26) or individual (n=30) format, and were often supervised (n=60) and delivered face-to-face (n=56). Duration ranged from 1 to 52 weeks and total contact time with the provider from 2 to 56 hours. Results of 42 interventions were suitable for meta-analysis. Univariate meta-regressions to evaluate associations between intervention characteristics and intervention effects directly after the intervention yielded no significant results. Due to self-reported outcomes and difficulties with blinding, risk of bias was high in all studies. To conclude, intervention characteristics were not associated with changes in FOF in this study. Possible reasons for an absence of associations and future research directions will be discussed.

